# Two-year dynamic risk stratification and adjuvant radioiodine efficacy in initial non-distant metastatic papillary thyroid carcinoma harboring TERT promoter mutations

**DOI:** 10.3389/fonc.2026.1895987

**Published:** 2026-09-30

**Authors:** Xian-Zhao Deng, Meng-yue Liu, Chen-Tian Shen, Hong-Jun Song, Jian Wang, You-Ben Fan, Quan-yong Luo, Juan Tang, Xiao-er Wei, Zhong-Ling Qiu

**Affiliations:** 1 Department of General Surgery, Sixth People’s Hospital Affiliated to Shanghai Jiao Tong University School of Medicine, Shanghai, China; 2 Department of Nuclear Medicine, Sixth People’s Hospital Affiliated to Shanghai Jiao Tong University School of Medicine, Shanghai, China; 3 Department of Pathology, Shanghai Sixth People’s Hospital Affiliated to Shanghai Jiao Tong University School of Medicine, Shanghai, China; 4 Institute of Diagnostic and Interventional Radiology, Shanghai Sixth People’s Hospital Affiliated to Shanghai Jiao Tong University School of Medicine, Shanghai, China

**Keywords:** effect, papillary thyroid cancer, progression-free survival, radioiodine therapy, TERT-p mutation

## Abstract

**Background:**

The clinical utility of postoperative radioiodine (^131^I) ablation remains unclear for patients with initially diagnosed non-distant metastatic papillary thyroid carcinoma (PTC) with harboring TERT promoter(TERT-p) mutations, it is unknown whether ^131^I therapy reduces the risk of persistent or recurrent disease. This study investigated clinicopathological features, 2-year dynamic therapeutic responses and progression-free survival (PFS) in this specific patient cohort.

**Methods:**

A single-center retrospective cohort study enrolled 113 eligible patients, allocated to the ^131^I ablation group (n=62) and non-^131^I group (n=51). Two-year dynamic risk stratification assessed treatment response. Kaplan–Meier curves and Cox regression models were used for survival analysis.

**Results:**

The predominant TERT-p variant was C228T (89.4%), and most patients carried concurrent BRAF^V600E^ mutations. Only initial surgical approach remained an independent intergroup variable. Within 2 years, 46.0% achieved acceptable responses, associated with sex and primary surgery (p=0.036,p < 0.001). High baseline ATA risk was strongly correlated with structural residual lesions and unfavorable therapeutic response (p < 0.001).No significant between-group differences in response were observed across all ATA risk subgroups. The median follow-up was 4.6 years; 3- and 5-year PFS rates were 67.5% and 39.1%. Age ≥55 years, N1b stage and high baseline ATA risk were independent adverse predictors of PFS. Both 2-year therapeutic response and PFS at the end of follow-up exhibited no statistical differences between the ^131^I group and non-^131^I group.

**Conclusions:**

In this retrospective observational cohort, postoperative ^131^I ablation was not associated with improved PFS among patients with non-distant metastatic TERT-p-mutant PTC. Advanced age, N1b lymphatic metastasis and high baseline ATA risk independently predict poor outcomes. Biologically, TERT-p mutations induce tumor dedifferentiation and impaired iodine uptake, diminishing ^131^I efficacy. Clinically, routine ^131^I yields no survival benefit for this high-risk molecular subgroup, guiding individualized surveillance and avoiding redundant ^131^I exposure.

## Introduction

Multiple malignant tumors, including thyroid carcinoma, are driven by oncogenic alterations such as BRAF, RAS, RET, NTRK fusions, and telomerase reverse transcriptase promoter (TERT-p) mutations (1–4). Two recurrent heterozygous C>T hotspot variants (C228T and C250T) within the TERT-p were first identified via whole-genome sequencing in melanoma; these two mutations are mutually exclusive (2, 3). TERT-p mutations constitute critical oncogenic events in thyroid cancer. These hotspot variants create novel binding sites for ETS transcription factors, markedly upregulate TERT transcription, activate telomerase, and promote tumor progression and dedifferentiation, ultimately leading to aggressive biological phenotypes and blunted treatment responsiveness (5).

Papillary thyroid carcinoma (PTC) is the most prevalent endocrine malignancy, with rapidly rising incidence across China and worldwide in recent decades (6). TERT-p mutations are frequently detected in aggressive PTC histologic variants, poorly differentiated thyroid cancer, and anaplastic thyroid cancer (7, 8). Within differentiated thyroid carcinoma (DTC), the prevalence of TERT-p mutations ranges from 10% to 20% across published cohorts (9). Mutant TERT-p is strongly linked to adverse clinicopathological features including advanced age, large tumor size, extrathyroidal extension, and distant metastasis, and serves as a robust molecular biomarker for elevated recurrence risk (10–14). Concurrent TERT-p and BRAFV600E mutations further amplify high-risk tumor phenotypes [13.14].

Postoperative radioiodine(^131^I) ablation has been a cornerstone of DTC management for nearly 80 years (15). For patients with distant metastatic DTC, two-thirds receive therapeutic ^131^I; half achieve durable remission, while the remaining one-third develop radioiodine refractoriness and experience early disease-related death (16). Multiple studies have confirmed TERT-p mutations as a biomarker of ^131^I resistance in distant metastatic DTC (17–21). For non-distant metastatic DTC, guideline-directed ^131^I ablation aims to reduce recurrence risk and prolong disease-free survival (15).

However, critical knowledge gaps remain specifically for patients with initially diagnosed non-distant metastatic PTC carrying TERT-p mutations. It remains unclear whether postoperative ^131^I can mitigate postoperative persistent or recurrent disease in this molecularly high-risk subgroup. Prior research primarily contrasts TERT-p mutant versus wild-type cohorts and validates the adverse pathological correlates of TERT-p mutations, yet few studies focus exclusively on non-distant metastatic PTC with TERT-p alterations. Whether these patients face uniform recurrence risks and comparable long-term prognosis cannot be reliably inferred from existing literature. Accordingly, this study comprehensively characterizes clinicopathological profiles, 2-year dynamic therapeutic responses to ^131^I, and progression-free survival (PFS) in a large cohort of patients with initial non-distant metastatic PTC and TERT-p mutations, to fill this unaddressed clinical evidence gap.

## Materials and methods

### Patients

We retrospectively reviewed clinical data from 7,476 patients who underwent thyroid surgery for PTC between January 2016 and December 2023 at the General Surgery and Nuclear Medicine Departments of Shanghai Jiao Tong University Affiliated Sixth People’s Hospital. A total of 2,354 patients received molecular testing for BRAFV600E, TERT-p, RAS, RET, and NTRK alterations using postoperative thyroid resection specimens. Among these patients, 132 harbored TERT-p mutations detected at initial diagnosis. Initial non-distant metastatic status was confirmed via preoperative chest computed tomography (CT), magnetic resonance imaging (MRI), 99mTc-MDP whole-body bone scan, or ^18^F-FDG PET/CT. We excluded 11 patients with synchronous distant metastases, eight patients with incomplete follow-up data, and one patient with concurrent breast cancer. The final study cohort included 113 patients with TERT-p-mutant PTC, stratified into an adjuvant ^131^I group (n=62) and a surgery-only non-^131^I group (n=51).

### TERT-p mutation analysis

TERT-p mutation status was detected by next-generation sequencing or Sanger sequencing. The next-generation sequencing method used was described in our previous report (22).The Sanger sequencing detection method was as follows. Genomic DNA was extracted from 5–10-µm-thick slices of formalin-fixed, paraffin-embedded primary PTC tissues using a QIAamp DNA FFPE Tissue Kit (Qiagen, Shenzhen, China) according to the manufacturer’s protocol (23).The regions harboring the spots in TERT-p C228T and C250T mutations were identified in genomic tumor DNA by polymerase chain reaction (PCR) and direct Sanger sequencing. 5′-AGTGGATTCGGGCACAGA-3′ (sense) and 5′-CAGCGTGCCTGAAACT-3′ (antisense) primers and 40–50 ng of genomic DNA were used to perform PCR amplification on the 235-bp region of TERT-p with C228T and C250T mutation hotspots.

### Initial thyroid surgery and adjuvant ^131^I therapy

In our series, 76 patients underwent total thyroidectomy combined with cervical lymph node dissection and 37 underwent unilateral lobectomy as the initial surgery. Sixty-two patients underwent ^131^I therapy in the months after their initial PTC surgery and during the follow-up. Among these 37 patients with unilateral lobectomy as the initial surgical method, 11 patients underwent the remaining lobectomy again and ^131^I treatment due to the persistence/recurrence during follow-up. The main reasons for not receiving ^131^I in 51 patients were as follows. First, ^131^I is generally not recommended in 26 patients undergoing unilateral lobectomy. Second, several studies have demonstrated a close association of TERT-p mutation in patients with DTC with refractoriness to ^131^I and reduced absorption of ^131^I. Therefore, some thyroid surgeons believe that ^131^I may be ineffective in 18 patients with PTC and TERT-p mutation and do not recommend it. The remaining seven patients have concerns about ^131^I causing secondary tumors and thus decline this treatment.

### Dynamic risk stratification at the first 2 years of follow-up

Dynamic therapeutic response was assessed within the first 2 years of postoperative follow-up per the 2025 American Thyroid Association (ATA) risk stratification framework (24). For patients receiving postoperative ^131^I ablation, four standardized response categories were adopted: excellent response, indeterminate response, biochemical incomplete response, and structural incomplete response. Excellent and indeterminate responses were collectively defined as acceptable therapeutic outcomes, while biochemical and structural incomplete responses were categorized as unacceptable outcomes (25). For patients who underwent thyroid surgery without adjuvant ^131^I therapy, we applied the Momesso dynamic response criteria tailored to lobectomy or total thyroidectomy cohorts without remnant ablation: an acceptable response corresponds to undetectable non-stimulated thyroglobulin (Tg) without suspicious neck imaging abnormalities, whereas elevated Tg or confirmed structural lesions indicate an unacceptable response (26).

### Definition of persistent/recurrent disease and follow-up

Disease progression was monitored by clinical examination, neck ultrasound, and measurement of serum thyroglobulin and thyroglobulin antibody concentrations. All patients underwent thyroid function testing (including serum T3, T4, and TSH, and thyroglobulin and thyroglobulin antibodies) every 3–6 months. Neck ultrasound was performed every 6–12 months, and some patients underwent CT, magnetic resonance imaging (MRI), and ^18^F-FDG PET/CT as necessary (27). Given the difficulty of distinguishing between recurrent and persistent disease in patients with a follow-up of less than 2 years (28). In this study, a lesion that was detected after the initial surgery was defined as persistent/recurrent disease. The final diagnosis of recurrent disease relies on ultrasonography, enhanced CT, MRI, ^18^F-FDG PET/CT, and/or pathological results. Progression-Free Survival (PFS) was defined as the interval between initial surgery and the first detection of persistent/recurrent disease or death from any cause.

### Statistical analysis

Continuous variables were first assessed for normality via the Shapiro–Wilk test. Normally distributed data were presented as mean ± standard deviation (SD) and compared by independent samples t-test; non-normal continuous data were reported as median (interquartile range, IQR) and compared using the Mann–Whitney U test. Categorical variables were summarized as counts and percentages, with between-group comparisons conducted via the χ^2^ test or Fisher’s exact test as appropriate. To adjust baseline confounding factors and mitigate baseline imbalance bias, we set the univariate screening cutoff at P < 0.2, and the retained variables were included in multivariate binary logistic regression models during the comparison of clinical characteristics between the ^131^I-treated and untreated groups. Univariate analyses using χ^2^ test or Fisher’s exact test were adopted to screen clinicopathological correlates of 2-year therapeutic response. For PFS analysis, Kaplan–Meier curves were plotted, and log-rank tests were used for simple intergroup survival comparison only for figure visualization. Comprehensive univariate Cox proportional hazards regression was performed for all clinicopathological variables to calculate hazard ratios (HR) and corresponding 95% confidence intervals (95% CI). All variables with P < 0.1 in univariate Cox analysis were incorporated into the multivariate Cox proportional hazards regression model to identify independent prognostic factors for PFS. All statistical analyses were carried out with MedCalc v17.0 and GraphPad Prism 7. A two-sided p-value < 0.05 was defined as statistically significant.

## Results

### Clinicopathological characteristics

A total of 113 patients with TERT-p mutation were included in the final analysis. The demographic and clinicopathological characteristics of the study cohort are shown in **Table 1**. Sixty-two cases (54.9%) received postoperative ^131^I and 51 (45.1%) did not. Total thyroidectomy was performed in 76(67.3%) patients and lobectomy in 37(32.7%) ones at the initial surgery. Among the 37 cases, eleven patients with persistent/recurrent PTC who underwent residual lobectomy again received ^131^I treatment during follow-up. The clinical characteristics and follow-up results of these patients were shown in **Supplementary Table S1**. When comparing the ^131^I treatment group and non‐^131^I treatment group, univariate analysis revealed significant differences in the initial surgical approach, number of surgical procedures, and multifocality (p<0.001, p=0.006, and p=0.009, respectively). Multivariate analysis further demonstrated that only initial surgical approach was independently associated with group allocation (p=0.01). No significant differences were observed for No. of surgeries, multifocality, extrathyroidal extension (all p>0.05) (**Table 1**).

**Table 1:** Baseline characteristics of patients with TERT promoter-mutant papillary thyroid carcinoma stratified by radioiodine ablation status.

Characteristic	Overall n (%)	Entire cohort (n =113 )		P value	Multivariate analyses		
		Non-^131^I(n=51) n (%)	^131^I(n=62) n (%)		OR	95%CI	P value
Age at initial diagnosis(years)				0.47			
<55	28(24.8)	11(9.7)	17(15.1)				
≥55	85(75.2)	40(35.4)	45(39.8)				
Median,mean(range)	62,61.98(37-89)	64,62.45(37-89)	60,61.5(37-79)				
Sex (F)				0.44			
F	62(54.9)	30(26.5)	32(28.4)				
M	51(45.1)	21(18.6)	30(26.5)				
The initial surgical procedure *				<0.001			
Total thyroidectomy	76(67.3)	25(22.1)	51(45.2)		1		
Lobectomy	37(32.7)	26(23.0)	11(9.7)		4.27	1.23-6.54	0.01
No. of surgeries				0.006			
1	75(66.4)	27(23.9)	48(42.5)		1		
>1	38(33.6)	24(21.2)	14(12.4)		2.11	0.53-4.62	0.06
Histopathology				0.70			
Classic PTC	60(53.1)	25(22.12)	35(30.97)				
Tall cell variant PTC	41(36.2)	21(18.58)	20(17.70)				
Hobnail-variant PTC	5(4.4)	2(1.77)	3(2.65)				
Tall cell+Hobnail variant PTC	3(2.6)	2(1.77)	1(0.88)				
Diffuse sclerosis variant PTC	4(3.5)	1(0.88)	3(2.65)				
Tumour size, (cm)				0.65			
<2	35(31.0)	18(15.9)	17(15.1)				
2-4	67(59.3)	28(24.8)	39(34.5)				
≥4	11(9.7)	5(4.4)	6(5.3)				
Multifocality				0.009			
1	67(59.3)	37(32.8)	30(26.5)		1		
>1	46(40.7)	14(12.3)	32(27.4)		1.83	0.67-2.72	0.15
Extrathyroidal extension				0.17			
Absent	75(66.4)	30(26.5)	45(39.8)		1		
Microscopic	10(8.8)	7(6.2)	3(2.6)		1.55	0.52-2.23	0.39
Gross	28(24.8)	14(12.4)	14(12.4)		1.74	0.69-2.51	0.30
T stage				0.90			
T1	25(22.1)	12(10.6)	13(11.5)				
T2	42(37.2)	17(15.0)	25(22.2)				
T3	21(18.6)	10(8.8)	11(9.8)				
T4	25(22.1)	12(10.6)	13(11.5)				
N stage				0.99			
N0	13(11.5)	6(5.3)	7(6.2)				
N1a	20(17.7)	9(8.0)	11(9.7)				
N1b	80(70.8)	36(31.9)	44(38.9)				
TERT mutational status				0.29			
TERT alone	7(6.1)	2(1.8)	5(4.4)				
TERT+ BRAF^V600E^	101(89.4)	45(39.8)	56(49.6)				
TERT+RET	2(1.8)	2(1.8)	0				
TERT+RAS	3(2.7)	2(1.8)	1(0.9)				
TERT mutation site				0.72			
C228T	101(89.4)	45(39.8)	56(49.6)				
C250T	12(10.6)	6(5.3)	6(5.3)				
ATA risk stratification				0.39			
Low	10(8.8)	7(6.2)	3(2.7)				
Intermediate-low	19(16.8)	9(8.0)	10(8.8)				
Intermediate-high	42(37.2)	17(15.0)	25(22.1)				
High	42(37.2)	18(15.9)	24(21.2)				
Dynamic risk stratification*							
Excellent	20(17.7)	11(9.7)	9(8.0)	0.33			
Indeterminate	32(28.3)	17(15.0)	15(13.3)				
Biochemical incomplete response	18(15.9)	8(7.1)	10(8.8)				
Structural incomplete response	43(38.1)	15(13.3)	28(24.8)				
Persistent/recurrent diseases				0.25			
No	62(54.9)	31(27.5)	31(27.4)				
Yes	51(45.1)	20(17.7)	31(27.4)				

TERT, telomerase reverse transcriptase; PTC, papillary thyroid carcinoma; RAI, radioiodine ablation; OR, odds ratio; CI, confidence interval; ATA, American Thyroid Association.

Categorical variables were compared by the χ^2^ test or Fisher’s exact test as appropriate. Binary logistic regression was used to screen independent factors associated with radioiodine ablation administration. Dynamic risk stratification: Risk stratification evaluated within the first 2 years after initial treatment.

### Genetic characterization

Genetic profiles of the TERT-p-positive cohort are summarized in **Table 1**. Among 2,354 patients with genetic testing, the overall TERT-p mutation prevalence was 4.80% (113 cases). Within the 113 TERT-p-mutant patients, mutations were detected via Sanger sequencing in 68 individuals (60.2%) and next-generation sequencing in 45 individuals (39.8%). C228T was the predominant TERT-p hotspot variant (101 patients, 89.4%), while C250T was identified in 12 patients (10.6%), corresponding to a C228T:C250T ratio of 8.42:1. Most patients harbored concurrent BRAF V600E plus TERT-p mutations (101, 89.4%); only 7 patients (6.1%) had isolated TERT-p mutations, 2 (1.8%) carried TERT-p with RET alterations, and 3 (2.7%) presented TERT-p combined with RAS mutations.

### Correlations of clinicopathological features with 2-year dynamic risk stratification

Dynamic risk stratification was performed based on clinical assessments conducted within the first 2 years of follow-up. Overall, 20 patients (17.7%) achieved an excellent response, 32 (28.3%) had an indeterminate response, 18 (15.9%) showed biochemical incomplete response, and 43 (38.1%) presented structural incomplete response (**Figure 1** and **Table 1**). In total, 52 patients (46.0%) attained acceptable treatment response, while the remaining 61 (54.0%) exhibited unacceptable response.

**Figure 1:**
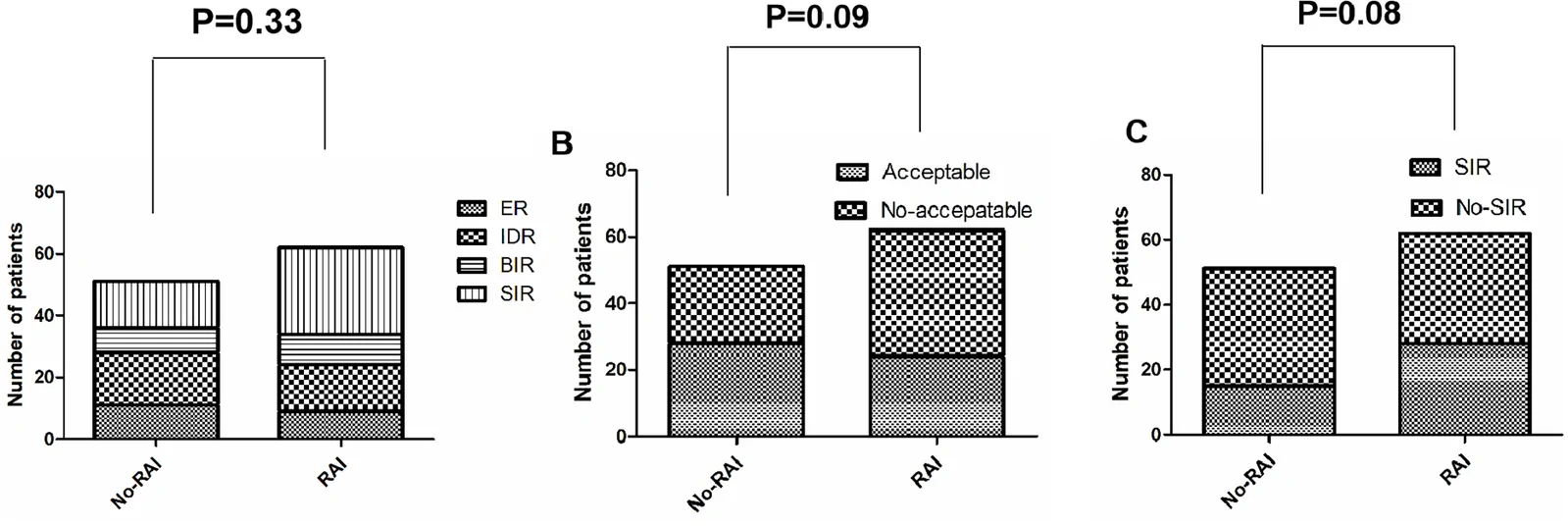
Two-year dynamic therapeutic response stratified by ^131^I treatment status. **(A)** Distribution of four response categories (excellent response, ER; indeterminate response, IDR; biochemical incomplete response, BIR; structural incomplete response, SIR) in patients with and without ^131^I treatment (P = 0.201). **(B)** Comparison of acceptable versus unacceptable treatment response between the non-^131^I and ^131^I groups (P = 0.09). **(C)** Comparison of structural incomplete response (SIR) and non-SIR between the non-^131^I and ^131^I cohorts (P = 0.08).

Among 62 patients receiving ^131^I therapy, 24 (21.2%) had acceptable response and 38 (33.6%) had unacceptable response; among 51 patients without ^131^I ablation, 28 (24.8%) achieved acceptable response and 23 (20.4%) showed unacceptable response (**Figure 1B** and **Table 2**). For structural incomplete response (SIR), 28 (24.8%) patients in the ^131^I group developed SIR and 34 (30.1%) did not; in the non- ^131^I group, SIR occurred in 15 (13.3%) patients and non-SIR in 36 (31.9%) patients (**Figure 1C**, and **Table 2**).

**Table 2:** Clinicopathologic factors for treatment response prediction in PTC patients with TERT-p mutations at the first 2 years of follow-up.

Characteristic	Acceptable response(n=52)n (%)		P-value	Structural incomplete response (n=43)(%)		P-value
	Yes	No		No	Yes	
Age at initial diagnosis(years)			0.63			0.23
<55	14(12.4)	14(12.4)		20(17.7)	8(7.1)	
≥55	38(33.6)	47(41.6)		50(44.2)	35(31.0)	
Sex (F)			0.04			0.35
Female	23(20.4)	39(34.5)		36(31.9)	26(23.0)	
Male	29(25.7)	22(19.5)		34(30.1)	17(15.0)	
The initial surgical procedure			<0.001			0.70
Total thyroidectomy	44(38.9)	32(28.3)		48(42.5)	28(24.8)	
Lobectomy	8(7.1)	29(25.7)		22(19.5)	15(13.3)	
No. of surgeries			0.32			0.53
1	37(32.7)	38(33.6)		48(42.5)	27(23.9)	
>1	15(13.3)	23(20.4)		22(19.5)	16(14.2)	
Histopathology			0.37			0.95
Classic PTC	30(26.5)	30(26.5)		37(32.7)	23(20.4)	
Aggressive variant of PTC	22(19.5)	31(27.4)		33(29.2)	20(17.7)	
T stage			0.75			0.85
T1-2	30(26.5)	37(32.7)		42(37.2)	25(22.1)	
T3-4	22(19.5)	24(21.2)		28(24.8)	18(15.9)	
Multifocality			0.14			0.17
Yes	27(23.9)	40(35.4)		38(33.6)	29(25.7)	
No	25(22.1)	21(18.6)		32(28.3)	14(12.4)	
Extrathyroidal extension			0.84			0.53
Absent	34(30.1)	41(36.3)		48(42.5)	27(23.9)	
Present	18(15.9)	20(17.7)		22(19.5)	16(14.2)	
N stage			0.36			0.14
N_0-1a_	13(11.5)	20(17.7)		17(15.0)	16(14.2)	
N_1b_	39(34.5)	41(36.3)		53(46.9)	27(23.9)	
TERT-p mutational status						0.01
TERT+ BRAF^V600E^	49(43.4)	52(46.0)		67(59.3)	34(30.1)	
TERT alone or combined with other mutations	3(2.7)	9(8.0)		3(2.7)	9(7.9)	
TERT-p mutational site			0.14			0.76
C228T	48(42.5)	53(46.9)		63(55.8)	38(33.6)	
C250T	4(3.5)	8(7.1)		7(6.2)	5(4.4)	
ATA risk stratification			0.51			<0.001
Low-Intermediate	31(27.4)	40(35.4)		57(50.4)	14(12.4)	
High	21(18.6)	21(18.6)		13(11.5)	29(25.7)	
^131^I treatment			0.09			0.08
Yes	24(21.2)	38(33.6)		34(30.1)	28(24.8)	
No	28(24.8)	23(20.4)		36(31.9)	15(13.3)	

Categorical variables were compared using χ^2^ test or Fisher’s exact test when appropriate. All percentages represent the proportion of the total cohort (n=113), rounded to one decimal place. Aggressive variant of PTC included tall cell variant (n=41), hobnail variant (n=5), combined tall cell + hobnail variant (n=3), and diffuse sclerosis variant (n=4).

TERT-p, TERT promoter; PTC, papillary thyroid carcinoma; SIR, structural incomplete response; ATA, American Thyroid Association; ^131^I, radioiodine-131.

Univariate regression analysis revealed that sex and primary surgical strategy were significantly correlated with 2-year acceptable/unacceptable response status (p = 0.036 and p < 0.001, respectively) (**Table 2**). Among patients carrying TERT-p mutations, the presence of structural lesions was strongly associated with baseline ATA risk stratification (p < 0.001).

### Association between baseline ATA risk stratification and 2-year dynamic risk stratification by ^131^I treatment status

We stratified patients by baseline ATA risk and compared AR/No-AR and SIR/non-SIR between ^131^I and non-^131^I cohorts (**Table 3**). Low and intermediate-low risk patients rarely developed No-AR or SIR in both groups. For intermediate-high and high-risk subgroups, counts of AR, No-AR, non-SIR and SIR were comparable between ^131^I and non-^131^I patients. No significant intergroup differences were detected in AR/No-AR distribution across all risk strata (p=0.30, 0.58, 0.47, 0.84), nor in SIR rates within intermediate-high (p=0.23) and high-risk subgroups (p=0.46).

**Table 3:** Association between ATA baseline risk stratification and 2-year dynamic treatment response stratified by radioiodine ablation status.

ATA risk stratification	N	Non-RAI (n=51)		RAI(n=62)		p	Non-RAI(n=51)		RAI(n=62)		p
		AR n(%)	No-AR n(%)	AR n(%)	No-AR n(%)		No-SIR n(%)	SIR n(%)	No-SIR n(%)	SIR n(%)	
Low	10	7(13.7)	0(0.0)	2(3.2)	1(1.6)	0.30	7(13.7)	0(0.0)	3(4.8)	0(0.0)	–
Intermediate-low	19	8(15.7)	1(2.0)	7(11.3)	3(4.8)	0.58	9(17.6)	0(0.0)	10(16.1)	0(0.0)	–
Intermediate-high	42	8(15.7)	9(17.6)	9(14.5)	16(25.8)	0.47	12(23.5)	5(9.8)	13(21.0)	12(19.4)	0.23
High	42	5(9.8)	13(25.5)	6(9.7)	18(29.0)	0.84	8(15.7)	10(19.6)	8(12.9)	16(25.8)	0.46
Total	113	28(54.9)	23(45.1)	24(38.7)	38(61.3)	0.09	36(70.6)	15(29.4)	34(54.8)	28(45.2)	0.08

RAI, radioiodine ablation; AR, acceptable response; SIR, structural incomplete response; ATA, American Thyroid Association.

Categorical variables were compared using the χ^2^ test or Fisher’s exact test as appropriate. All percentages represent the proportion of patients in the corresponding Non-RAI/RAI group, rounded to one decimal place.

### Association between PFS rate and clinicopathological characteristics

By the end of follow-up, persistent/recurrent disease was detected in 51 patients (45.1%) Diagnostic modalities used to detect persistent/recurrent disease were showed in the **Supplementary Table S2** and **Figure 2**. The 113 patients were followed up for a median of 4.6 years (range, 0.1–6.9), during which 51 (43.97%) developed recurrence. At the end of follow-up, 105 patients were alive, and 8 (7.80%) had died of persistent/recurrent disease. The 3-year and 5-year cumulative PFS rates were 67.5% and 39.1%, respectively (**Figure 3A**).

**Figure 2:**
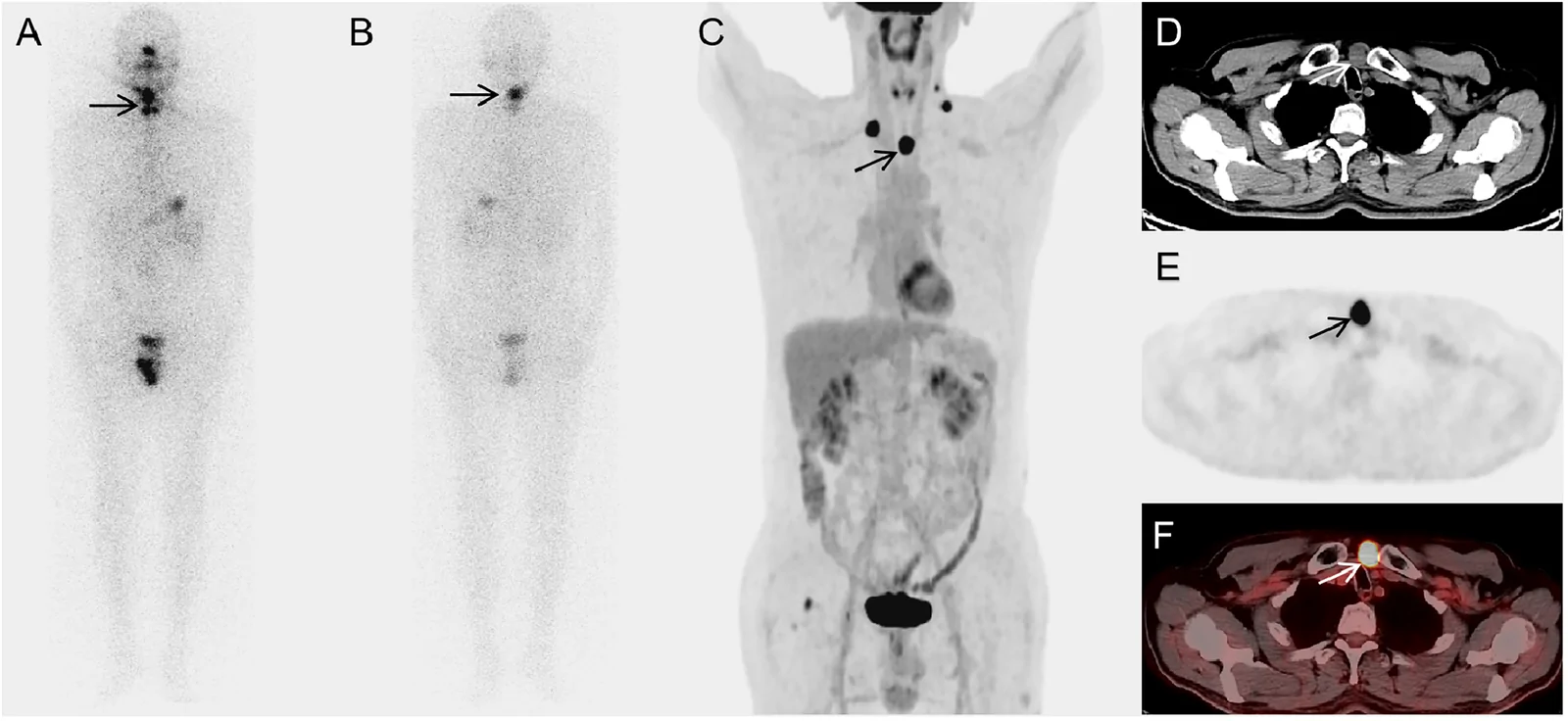
A 53-year-old male with TERT-p/BRAFV600E-mutant tall cell variant PTC received total thyroidectomy plus central neck dissection in June 2021, followed by adjuvant ^131^I ablation after 4 weeks of low iodine diet and levothyroxine withdrawal. Pre-treatment cervical ultrasound was unremarkable; stimulated Tg, TgAb and TSH were 91 ng/mL, 11.5 KIU/L and 57.2 mIU/L, respectively. Post-ablation ^131^I-WBS [anterior **(A)**, posterior **(B)**] at day 5 after 5.55 GBq oral ^131^I only showed intense uptake in thyroid remnant (black arrow). At 2.2-year follow-up, TSH-suppressed Tg rose gradually from 8.2 to 25.4 ng/mL. Subsequent ^18^F-FDG PET/CT [MIP image **(A)**] detected multiple neck metastatic lymph nodes with SUVmax 9.6 [black arrows in **(D–F)**], which were verified as PTC metastases on postoperative pathology.

**Figure 3:**
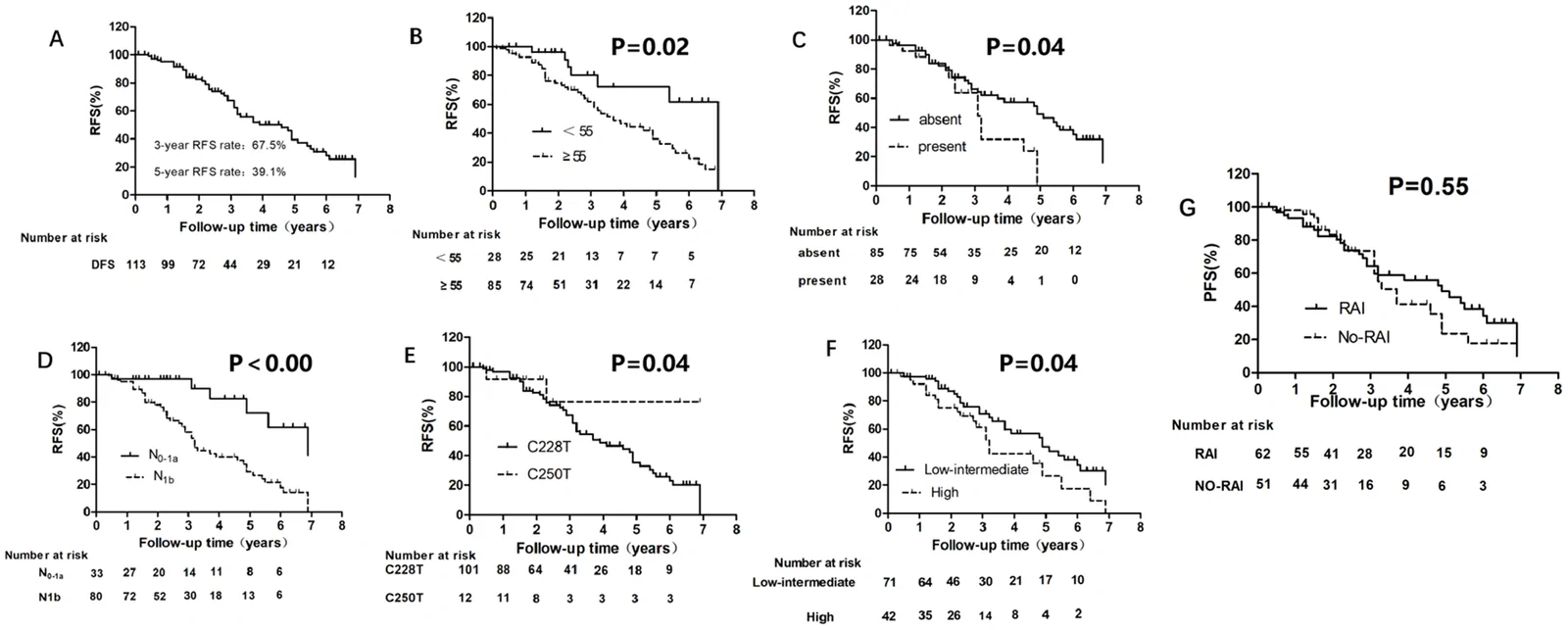
Kaplan–Meier survival curves for progression-free survival (PFS) among 113 PTC patients with TERT-p mutations. The 3- and 5-year cumulative PFS rates were 67.5% and 39.1%, respectively. Significant differences in PFS were observed stratified by age [≥55 vs. <55 years, **(B)**], extrathyroidal extension status **(C)**, N stage [N0–1a vs. N1b, **(D)**], TERT mutation site [C228T vs. C250T, **(E)**], and ATA risk tier [low-intermediate vs. high, **(F)**].

Log-rank test demonstrated that the age at initial diagnosis, extrathyroidal extension, N stage, TERT-p mutation site, and ATA risk stratification were significantly associated with the PFS rate (p=0.017, p=0.047, p<0.001, p=0.040, and p=0.036, respectively) (**Figures 3B–F**). However, there was no significant association between the PFS rate and any of the remaining factors including ^131^I treatment (p>0.05) (**Table 4**, **Figure 3G**).

**Table 4:** Log-rank test, univariate and multivariate Cox proportional hazards regression analyses of prognostic factors for progression-free survival in PTC patients with TERT promoter mutations.

	Log-rank test				Cox regression analyses					
Characteristic	N	No.PFS ( N%)	χ^2^	P	Univariate analyses			Multivariate analyses		
					HR	95%CI	P	HR	95%CI	P
Age at initial diagnosis(years)			5.68	0.02						
<55	28	7(25.0)			1			1		
≥55	85	44(51.8)			2.36	1.37-4.86	0.01	2.29	1.18-4.44	0.02
Sex (F)			0.73	0.40						
Female	62	27(43.5)			1					
Male	51	24(47.1)			1.51	0.88-2.58	0.14			
Initial surgical procedure			0.65	0.32						
Total thyroidectomy	76	32(42.1)			1					
Lobectomy	37	19(51.4)			1.07	0.71-1.89	0.76			
No. of surgeries			0.21	0.65						
1	75	23(30.7)			1					
>1	38	28(73.7)			1.36	0.68-1.93	0.54			
Tumour size			0.41	0.81						
<2	35	16(45.7)			1					
2-4	67	33(49.2)			1.23	0.83-1.84	0.30			
≥4	11	2(18.2)			1.69	0.76-3.74	0.22			
Histopathology			0.77	0.38						
Classic	60	33(55.0)			1					
variants	53	18(34.0)			1.82	0.59-2.77	0.43			
T stage			0.31	0.58						
T1-2	67	34(50.7)			1					
T3-4	46	17(37.0)			1.50	0.89-2.53	0.12			
Multifocality			0.33	0.57						
1	67	27(40.3)			1					
>1	46	24(52.2)			1.68	0.44-2.96	0.49			
Extrathyroidal extension			3.93	0.05						
Absent	75	37(49.3)			1			1		
Present	28	14(50.0)			2.21	1.56-2.45	0.04	1.62	0.93-2.04	0.09
N stage			12.97	<0.001						
N_0-1a_	33	6(18.2)			1			1		
N_1b_	80	45(56.3)			3.23	1.46-5.82	<0.001	3.72	1.25-5.62	<0.001
TERT mutational status			1.53	0.22						
TERT+ BRAF^V600E^	101	46(45.5)			1					
TERT alone or combined with other mutations	12	5(41.7)			1.47	0.66-3.37	0.35			
TERTp mutational site			4.22	0.04						
C228T	101	49(48.5)			1			1		0.50
C250T	12	2(16.7)			0.47	0.21-1.07	0.07	0.63	0.34-3.88	
ATA risk stratification			4.39	0.04						
Low-Intermediate	71	29(40.8)			1			1		
High	42	22(52.4)			2.38	1.46-3.41	0.03	2.51	1.39-3.72	0.04
^131^I treatment			0.35	0.55						
Yes	62	30(48.4)			1					
No	51	21(41.2)			1.46	0.88-2.42	0.15			

PTC, papillary thyroid carcinoma; TERT, telomerase reverse transcriptase; PFS, progression-free survival; HR, hazard ratio; CI, confidence interval; ATA, American Thyroid Association; ¹³¹I, radioiodine-131.

Log-rank test was used to compare progression-free survival between subgroups; univariate and multivariate Cox proportional hazards regression models were applied to screen prognostic factors.Variables with P < 0.05 in univariate analysis were enrolled into multivariate model. Ref., reference group. All percentages represent the proportion of progression events within each subgroup, rounded to one decimal place.

Univariate Cox regression revealed that age ≥55 years (HR = 2.36, 95%CI 1.37–4.86, P = 0.01), extrathyroidal extension (HR = 2.21, 95%CI 1.56–2.45, P = 0.04), N1b metastasis (HR = 3.23, 95%CI 1.46–5.82, P<0.001), high ATA risk (HR = 2.38, 95%CI 1.46–3.41, P = 0.03) were associated with shorter PFS. Variables with P < 0.1 in univariate Cox regression were entered into the multivariate Cox proportional hazards regression model. Multivariate analysis identified three independent poor prognostic factors: age ≥55 years (HR = 2.29, 95%CI 1.18–4.44, P = 0.02), N1b metastasis (HR = 3.72, 95%CI 1.25–5.62, P<0.001), high ATA risk (HR = 2.51, 95%CI 1.39–3.72, P = 0.04). Extrathyroidal extension lost statistical significance after adjustment (HR = 1.62, 95%CI 0.93–2.04, P = 0.09) (**Table 4**).

## Discussion

This single-center retrospective cohort study systematically analyzed clinicopathological characteristics, 2-year DRS outcomes, and PFS in the end of follow-up in patients with initially diagnosed non-distant metastatic PTC harboring TERT-p mutations. Unlike most previous studies that compared TERT-p mutant and wild-type PTC, this work specifically focuses on the homogeneous molecular subgroup with TERT-p alterations without distant metastasis at baseline, providing targeted real-world evidence for postoperative ^131^I management and prognostic stratification.

Reports on the frequency of TERT-p mutation in PTC range widely in the literature from 2% to 61%, but are mainly concentrated in the range of 10%–20% (9). Most previous studies were small case series, which may show deviations in these results. The largest sample analyzed to date was in a study by Ebina et al (29),who reviewed 685 patients with PTC, 133 (19.4%) of whom had TERT-p mutations; the incidence rate of TERT-p mutation in our study was 4.8%, which is similar to that in the reports by Sun et al. (4.4%) and Liang et al. (2.0%) in China (30, 31);however, all these rates are significantly lower than those in other regions and Western countries (9). The reasons for the discordant findings may reflect differences in factors such as race, sample size, prevalence of genetic testing, the proportion of patients with invasive PTC, the proportion with distant metastases, tumor risk stratification and staging, and the detection methods used (9).

Two sequencing platforms were utilized to identify TERT-p mutations in this retrospective cohort. NGS was prioritized for FFPE specimens with low tumor cellularity or limited DNA yield, while Sanger sequencing was adopted for samples with sufficient tumor tissue to reduce economic costs. Owing to the relatively lower analytical sensitivity of Sanger sequencing compared with NGS, the overall TERT-p mutation prevalence of 4.80% calculated from all screened PTC patients may underestimate the true mutation frequency. Differences in detection modalities represent an inherent limitation of this single-center retrospective analysis. Future prospective studies using a unified sequencing platform are required to verify our findings.

DRS is a validated framework to monitor postoperative disease evolution. The 2025 ATA guidelines recommend standardized assessment within the first two postoperative years as a powerful predictor of long-term recurrence risk (32, 33). To date, only Kim et al. have evaluated TERT-p status alongside DRS among non-distant metastatic differentiated thyroid cancer (DTC) (34). They identified divergent long-term PFS across response tiers but did not stratify patients by baseline ATA risk or adjuvant ^131^I treatment. Our study fills this evidence gap via standardized 2-year DRS assessment in 113 TERT-p-mutant non-metastatic PTC patients. Overall, acceptable responses were achieved in 46.0% of patients, while 54.0% had unacceptable responses. Acceptable response and structural incomplete response rates were comparable between patients receiving ^131^I and those treated with surgery alone. Further stratified analysis according to baseline ATA risk demonstrated that low and intermediate-low risk patients rarely developed adverse outcomes regardless of radioiodine administration. Among intermediate-high and high-risk patients, the distribution of acceptable/unacceptable responses and structural incomplete response still showed no significant intergroup differences (all p>0.05). Univariate analysis confirmed that high baseline ATA risk, rather than postoperative ^131^I ablation, was strongly associated with unfavorable short-term therapeutic outcomes and structural residual or recurrent disease.

We further evaluated long-term oncological outcomes in this TERT-p-mutant cohort. Published recurrence rates among TERT-p-positive PTC vary substantially (**Supplementary Table S3**), attributable to heterogeneous enrollment criteria, follow-up duration and ethnic composition (13, 35–38). In our cohort with a median follow-up of 4.6 years, the overall recurrence rate reached 45.1%, while the 3-year and 5-year cumulative PFS rates were 67.5% and 39.1%, respectively. Multivariate Cox regression identified age ≥55 years, lateral lymph node metastasis (N1b), and high baseline ATA risk stratification as independent negative prognostic factors for PFS, consistent with prior studies reporting worse outcomes in older patients with TERT-p-mutant DTC (11, 12, 39). Notably, primary tumor size, extrathyroidal extension and aggressive histologic subtypes lost independent prognostic significance. This inconsistency with earlier literature is likely explained by our strict enrollment criteria: all patients carried TERT-p mutations and presented without distant metastasis at baseline, whereas most published series combined TERT-p wild-type and mutant cases with mixed metastatic status (37, 38).

Postoperative ^131^I ablation is standard adjuvant therapy for advanced, persistent, recurrent or metastatic DTC. Multiple studies indicate that TERT-p mutations predict impaired iodine avidity and radioiodine refractoriness, yet most investigations focused on patients with established distant metastases (17–25). Evidence for newly diagnosed non-distant metastatic TERT-p-mutant PTC remains scarce, forming the core research question of this work: whether adjuvant ^131^I reduces short-term persistent disease risk or improves long-term PFS. Among the 113 patients, 62 received adjuvant ^131^I and 51 underwent surgery alone. After adjusting for baseline clinicopathological confounders, multivariate logistic regression revealed no significant between-group difference in 2-year DRS outcomes. Consistently, stratified analyses across ATA risk tiers and Kaplan–Meier/Cox models confirmed that adjuvant ^131^I exerted no independent impact on long-term PFS. These results should be interpreted cautiously and cannot be directly translated into routine clinical recommendations.

This single-center retrospective study has several limitations. First, non-randomized radioiodine assignment based on physician preference and patient refusal caused significant selection bias, which may obscure the true efficacy of ^131^I. Though multivariate regression adjusted for available clinical confounders, residual bias persisted from unrecorded subjective judgments, patients’ psychological worries, limited non-131I sample size and delayed salvage radioiodine. Thus, our retrospective findings await further validation in prospective studies. Second, we adopted both Sanger sequencing and NGS for TERT-p detection; the lower sensitivity of Sanger sequencing may underestimate the true mutation prevalence and introduce analytical bias. Third, the median follow-up was merely 4.6 years, which is insufficient to draw solid conclusions on long-term progression-free survival for this high-risk cohort. Fourth, all subjects were restricted to initially non-distant metastatic TERT-p-mutant PTC patients, so the results cannot be generalized to patients with distant metastasis or TERT wild-type tumors. Incomplete recording of individualized adjuvant therapies may also interfere with outcome assessment. Moreover, few patients had isolated TERT-p mutation without BRAF V600E, making meaningful subgroup analysis unfeasible, which is also a critical limitation.

## In conclusion

Among 113 newly diagnosed non-distant metastatic TERT-p-mutated PTC patients, 89.4% harbored concurrent TERT-p and BRAF V600E mutations. Only 46.0% achieved acceptable 2-year therapeutic responses according to the 2025 ATA dynamic risk stratification framework. After adjusting for clinicopathological confounders, 2-year treatment responses were comparable between patients receiving adjuvant ^131^I and those treated with surgery alone across all ATA risk subgroups, and adjuvant ^131^I exhibited no independent correlation with PFS. Multivariate Cox regression analysis identified age ≥55 years, N1b lymph node metastasis, and high baseline ATA risk stratification as independent adverse prognostic factors for shorter PFS. Although no intermediate-term PFS benefit from adjuvant ^131^I was observed in this cohort, this preliminary conclusion requires cautious interpretation, considering the retrospective single-center design, inconsistent genotyping detection methods, and limited sample size. Larger prospective multicenter studies are necessary to validate our findings. Clinicians may utilize these prognostic risk markers to formulate intensified postoperative surveillance strategies for this molecular subgroup.

## Data Availability

The original contributions presented in the study are included in the article/**Supplementary Material**. Further inquiries can be directed to the corresponding author.
