# Supplementary material for: Two-year dynamic risk stratification and adjuvant radioiodine efficacy in initial non-distant metastatic papillary thyroid carcinoma harboring TERT promoter mutations
Source: Front Oncol. 2026 Sep 30;16:1895987. doi: 10.3389/fonc.2026.1895987 (PMC13661339; doi:10.3389/fonc.2026.1895987)
Supplement: Supplementary file 1 [file Table1.docx]

**Supplementary Table S1: Baseline and recurrence-related clinical features of 11 PTC patients receiving initial lobectomy followed by ¹³¹I ablation**

| **Patient No** | **Sex** | **Ageat diagnosis** | **Initial surgery** | **Histopathology** | **Tumour size(cm)** | **Extrathyroidal extension** | **N stage** | **Recurrence time** | **Sites of recurrent diseases** |
| --- | --- | --- | --- | --- | --- | --- | --- | --- | --- |
| 1 | F | 57 | Lobectomy | High-cell subtype | 0.6 | - | N0 | 5.7 | CLM |
| 2 | M | 53 | Lobectomy+pICNS | Classic | 2.4 | - | N1a | 2.2 | CLM |
| 3 | F | 59 | Lobectomy+pICNS | Classic | 2.9 | - | N1a | 3.4 | CLM |
| 4 | F | 55 | Lobectomy | High-cell subtype | 1.2 | - | N0 | 2.7 | CLM+lungs |
| 5 | M | 60 | Incomplete surgery | Classic | 1.8 | Tracheal invasion | N1a | 0.9 | CLM+tracheal invasion |
| 6 | F | 49 | Lobectomy+pICNS | Classic | 1.9 | - | N0 | 5.1 | CLM |
| 7 | F | 64 | Lobectomy | High-cell subtype | 0.4 | - | N0 | 0.7 | CLM |
| 8 | F | 67 | Lobectomy+pICNS | High-cell subtype | 3.2 | - | N1a | 5.6 | Lungs |
| 9 | F | 56 | Lobectomy+pICNS | High-cell subtype | 1.9 | - | N1a | 4.9 | CLM+esophageal invasion |
| 10 | M | 53 | Lobectomy+pICNS | Classic | 2.6 | - | N1a | 3.4 | CLM+tracheal invasion |
| 11 | F | 46 | Lobectomy | Classic | 2.2 | - | N1a | 5.2 | lungs |

Age:age of initial diagnosis; Recurrence time: Recurrence time after initial surgery (years). pICND: prophylactic ipsilateral central neck dissection(IV).CLM:Cervical lymph node.
