# Supplementary material for: Two-year dynamic risk stratification and adjuvant radioiodine efficacy in initial non-distant metastatic papillary thyroid carcinoma harboring TERT promoter mutations
Source: Front Oncol. 2026 Sep 30;16:1895987. doi: 10.3389/fonc.2026.1895987 (PMC13661339; doi:10.3389/fonc.2026.1895987)
Supplement: Supplementary file 2 [file Table2.docx]

| **Sites of persistent/recurrent lesions** | **Diagnostic modality** | **No. of patients N(%)** |
| --- | --- | --- |
| **Cervical lymph node metastases** | neck ultrasonography +pathology | 10(9.6) |
|  | ^131^I-WBS+^131^I-SPECT/CT | 3(5.9) |
|  | ^18^F-FDG-PET/CT | 7(13.7) |
| **Mediastinal lymph node metastases** | ^18^F-FDG-PET/CT | 3(5.9) |
| **Local invasion** |  |  |
| Striated muscle invasion | enhanced MRI+ pathology. | 3(5.9) |
| Tracheal invasion | enhanced CT +pathology, | 8(15.7) |
|  | ^18^F-FDG-PET/CT | 4(7.8) |
| Esophageal invasion | enhanced CT | 2(3.9) |
|  | enhanced MRI | 2(3.9) |
| **Distant metastases** |  |  |
| Lung metastases | elevated Tg + chest CT | 10(19.6) |
|  | ^131^I-WBS +^131^I-SPECT/CT | 2(3.9) |
|  | ^18^F-FDG-PET/CT | 5(9.8) |
| Bone metastases | ^18^F-FDG-PET/CT | 3(5.9) |
|  |  |  |

Supplementary Table S2**：**Diagnostic modalities for persistent/recurrent lesions in 50 patients during follow-up

Abbreviations: RAI, radioiodine ablation; Tg, thyroglobulin; WBS, whole-body scan; SPECT/CT, single-photon emission computed tomography combined with computed tomography; MRI, magnetic resonance imaging; ¹⁸F-FDG-PET/CT, ¹⁸F-fluorodeoxyglucose positron emission tomography combined with computed tomography.
