# Supplementary material for: Two-year dynamic risk stratification and adjuvant radioiodine efficacy in initial non-distant metastatic papillary thyroid carcinoma harboring TERT promoter mutations
Source: Front Oncol. 2026 Sep 30;16:1895987. doi: 10.3389/fonc.2026.1895987 (PMC13661339; doi:10.3389/fonc.2026.1895987)
Supplement: Supplementary file 3 [file Table3.docx]

**Supplementary Table S3: Summary of key cohort studies reporting recurrence risk in TERT-p-mutant papillary thyroid carcinoma**

| **Reference (Year, Author)** | **Number of TERT-p positive PTC patients** | **Median/Mean follow-up duration** | **Recurrence/persistence cases & rate** | **Core cohort inclusion feature** | **Main discrepancy with our study** |
| --- | --- | --- | --- | --- | --- |
| Xing et al., 2014 [13] | 61 | Median 24 months | 29 cases, 46.81% 3-year recurrence rate | Mixed PTC cohort (TERT wild-type + mutant, including distant metastatic cases) | Enrolled all PTC without restricting to non-distant metastatic population; shorter follow-up, lower long-term recurrence risk than our data |
| Trybek et al., 2019 [35] | 77 | Mean 120 months | 8 cases, 10.38% | Unselected PTC cohort, mixed risk and metastatic status | Longest follow-up but far lower recurrence rate; study population contained fewer high-risk N1b patients |
| Póvoa et al., 2019 [36] | 16 | 10-year follow-up | 7 cases, 43.75% | Single-center general PTC cohort, small TERT-mutant sample size | Limited TERT-positive sample (n=16), no stratified analysis for non-distant metastatic subgroup |
| Vianello et al., 2021 [37] | 27 | Median 81.8 months | 8 cases, 66.7% adverse outcome (persistent/recurrent/death) | Stratified by tumor size, included distant metastatic DTC | Contained patients with distant metastasis at baseline, leading to higher composite adverse event rate |
| Song et al., 2016 [38] | 25 | Median 4.8 years | 7 cases, 28.0% | All differentiated thyroid cancer (PTC+FTC), mixed metastatic status | ncluded follicular thyroid carcinoma; lower recurrence rate than our pure non-distant metastatic PTC cohort |
| Present study (Current work) | 113 | Median 4.6 years (0.1–6.9 years) | 51 cases, 45.13% total recurrence; 3-year PFS=67.5%, 5-year PFS=39.1% | Strictly restricted to initially non-distant metastatic PTC with TERT-p mutation | xclusively TERT-mutant non-metastatic PTC; multivariate analysis identified age ≥55, N1b, high ATA risk as independent poor prognostic factors |
